# News and Miscellany

**Published:** 1905-02

**Authors:** 


### News and Miscellany.

'Tis not absinthe makes the heart grow fonder, 'tis Coca. (Vin Mariana.)

The Mexican International Railroad is building a $50,000 hospital at Puebla. Dr. T. H. Harrell, late of Hutto, Texas, will have charge of it.

Dr. J. T. Barnard, late resident physician of the Llano Sanitarium, is no longer connected with that institution. M. M. Smith, Superintendent.

"The cleanest medical journal that comes to my table."

C. S. Bobo, M. D.,
Norman, Okl.

Dr. T. J. Bennett of Austin suffered a fracture of the left femur (inter-capsular) on the 29th of January by being thrown from his auto, which turned over on him. He is doing as well as possible at this writing.

Wanted—A bright, up-to-date young doctor as assistant. Can at once enter good paying practice in German town and country. References required. I want to see applicant before making contract. Address "M," care *Texas Medical Journal.*

Here's to the doctor! a man of lore
Who knows the why and how to do;
He'll not forsake one at death's door,
Nor leave until he pulls you through (death's door?).

Wanted—To correspond with expert stenographer; capable of reporting medical society meetings.
Fort Worth, Texas. I. C. Chase, M. D.,
Secretary State Medical Association of Texas.

The Alpha Sanitarium for Tuberculosis, located for some years at De Soto, Texas, has been reopened at Llano, with J. T. Bernard, M. D., resident physician, and Drs. E. D. Mabey, C. F. Darnell, H. S. Selman, E. D. Townsend and A. Sacks. See card.

The House of Delegates of the State Medical Association and the Board of Medical Councilors and the Committee on Public Policy and Legislation met in Austin in extra session upon call of the President February 1st inst. It would be premature to inform our readers of the action of those bodies.

---

"This year we will do less medical journal advertising than for two decades past. If we could advertise in only two medical journals the Texas Medical Journal would be one of them."

Sharp & Dohme, New York.

This, after twenty years' continuous patronage. *Verbum sat sap.*

---

J. S. Tyree, the Chemist, Washington, D. C., sends us an ornamental card suspended by silk cords, on which is a match striker stamped "Scratch the world and you won't find anything better than Tyree's Antiseptic Powders." Many Texas doctors will remember Mr. Tyree when he was a "detail man" in Texas, and will be glad to know that he is now wholesale, and doing well on his own hook.

---

One of the Elect (exempt under Texas law):

Samuel Mackintosh (Voodoo Sam, Colored),
Physician and Surgeon, ——, Texas.
Offers his services to the people of —— for the treatment and cure
of all diseases and injuries without drugs.
No cure, no pay.
Can be found night and day at ——

"Doctor" Mackintosh can neither read nor write.—Daniel.

---

The Japs Put Us to Shame.—An Associated Press telegram from Oku's headquarters January 29th ult. says that the Chief Surgeon of Oku's army—approximately 100,000 men—reports officially that there have been in the entire army since landing, May 6th, only forty deaths from diseases. Up to December there were treated 24,642 cases. There were 193 cases of typhoid fever, 34 of dysentery, and 5079 of beriberi. These figures are unequaled in the history of warfare. Per contra, during the Spanish-American war 286 of our men died from bullets and wounds and 3862 died from preventable diseases. These are U. S. Surgeon Seaman's figures. And yet, and yet, we in Texas can not interest our law makers in the subject of sanitary science in relation to the public health.

---

New Subscribers since Last Issue (not Renewals): Dr. C.

A. H. Arnecke, Arneckeville, Texas; Dr. W. H. Allen, Marlin, Texas; Dr. A. Alexander, El Paso, Texas; Dr. R. B. Bell, Roddy, Texas; Dr. Z. T. Bundy, Millford, Texas; Dr. A. R. Bowman, Uvalde, Texas; Dr. J. B. Burditt, Brenham, Texas; Dr. C. S. Behrens, Cherokee, Texas; Dr. W. N. Brooks, Oletha, Texas; Dr. J. A. Burke, Cathon, Texas; Dr. J. T. Bernard, Llano, Texas; Dr. Price Cheeney, Dallas, Texas; Dr. W. L. Crossthwait, Holland, Texas; Dr. S. A. Collom, Ratcliff, Texas, Dr. J. F. Corry, Rockwall, Texas; Dr. A. J. Childress, Gilmer, Texas; Dr. R. T. Canon, Moscow, Texas; Dr. Russell Caffery, San Antonio, Texas; Dr. E. H. Cary, Dallas, Texas; Dr. J. J. Dial, Sulphur Springs, Texas; Dr. J. K. Davidson, Eagle Lake, Texas; Dr. J. E. Dodson, Vernon, Texas; Dr. G. W. Dukes, Lafayette, Texas; Dr. C. W. Echols, Bailey, Texas; Dr. J. H. Erwin, Tolosa, Texas; Dr. J. F. FitzSimon, Castroville, Texas; Dr. W. G. Fowler, Valley Springs, Texas; Dr. J. K. P. Green, Rancho, Texas; Dr. M. R. Hughes, St. Louis, Mo.; Dr. T. H. Harrell, Pueblo, Mexico; Dr. B. F. Jones, Cisco, Texas; Dr. L. Jankafsky, Pittsburg, Texas; Dr. J. D. Jackson, Tarkington, Texas; Dr. J. R. Knight, Eola, Texas; Dr. H. S. Kirby, Sigsbee, Texas, Eli, Lilly & Co., Indianapolis, Ind.; Dr. C. W. Legrand, Hempstead, Texas; Dr. J. T. Moore, Galveston, Texas; Dr. T. R. Moorehead, Ben Franklin, Texas; Dr. F. P. Miller, El Paso, Texas; Dr. C. W. McBurnett, Minerva, Texas; Dr. J. O. McReynolds, Dallas, Texas; Dr. O. O'Bar, St. Louis, Mo.; Dr. P. F. Robertson, Cartel, Texas; Dr. A. J. Rush, Paris, Texas; Dr. O. H. Radkey, Edna, Texas; Dr. L. H. Reeves, Newark, Texas; Dr. G. S. Stell, Paris, Texas; Dr. K. H. Seymour, Warrenton, Texas; Dr. T. S. Slater, Re, Texas; Dr. C. C. Shell, Stamford, Texas; Dr. G. W. Southern, Lincoln, Texas; Dr. J. W. Smith, Waukegan, Texas; Dr. M. J. Taylor, Camden, Texas; Dr. C. C. Taylor, Cooper, Texas; Dr. J. S. Turner, Terrell, Texas; Dr. J. C. Winn, Simpsonville; Dr. J. M. Ware, Magnolia, Texas; Dr. J. M. Weston, Sutherland Springs, Texas; Dr. C. R. Watkins, Floresville, Texas; Dr. R. H. Watkins, Amphion, Texas; Dr. W. C. Welch, Caddo Mills, Texas; Dr. C. M. Harrison, Cooper, Texas; Dr. E. T. Clarke, Keltys, Texas.

